# Correction: Pharmacological targeting of the transcription factor SOX18 delays breast cancer in mice

**DOI:** 10.7554/eLife.90408

**Published:** 2023-08-08

**Authors:** Jeroen Overman, Frank Fontaine, Mehdi Moustaqil, Deepak Mittal, Emma Sierecki, Natalia Sacilotto, Johannes Zuegg, Avril AB Robertson, Kelly Holmes, Angela A Salim, Sreeman Mamidyala, Mark S Butler, Ashley S Robinson, Emmanuelle Lesieur, Wayne Johnston, Kirill Alexandrov, Brian L Black, Benjamin M Hogan, Sarah De Val, Robert J Capon, Jason S Carroll, Timothy L Bailey, Peter Koopman, Ralf Jauch, Matthew A Cooper, Yann Gambin, Mathias Francois

**Keywords:** Mouse, Zebrafish

Overman J, Fontaine F, Moustaqil M, Mittal D, Sierecki E, Sacilotto N, Zuegg J, AAB, Robertson, Holmes K, Salim AA, Mamidyala S, Butler MS, Robinson AS, Lesieur E, Johnston W, Alexandrov K, Black BL, Hogan BM, De Val S, Capon RJ, Carroll JS, Bailey TL, Koopman P, Jauch R, Cooper MA, Gambin Y, Francois M. 2017. Pharmacological targeting of the transcription factor SOX18 delays breast cancer in mice. *eLife* **6**:e21221. doi: 10.7554/eLife.21221.

To ensure transparency, maintain integrity of the public record and fulfil our obligations of Responsible Conduct of Research, we hereby provide a correction to this paper.

Following a research integrity investigation by QIMR Berghofer Medical Research Institute, Brisbane, Australia (QIMRB), into papers containing experiments conducted by Professor Mark Smyth at QIMRB, it was concluded that both parts of Figure 4—figure supplement 1 were based on fabricated data.

One figure that was believed to be based on fabricated data, Figure 4—figure supplement 1, has been clearly identified. Accordingly, Figure 4—figure supplement 1 has been removed from the paper.

The dataset in the paper comprises 4 main figures and 11 figure supplements. Figure 4**—**figure supplement 1 is the only figure which has been found to be based on fabricated data. However, it contributed only minimally to the large body of data, addressing a minor mechanistic point. Moreover, Figure 4E also establishes the result previously shown in Figure 4**—**figure supplement 1. No other figures in the paper flowed from or were reliant on the result shown in the figure in question. Removal of Figure 4**—**figure supplement 1 has no effect on the study’s outcomes, as reflected in the title and abstract.

This correction notice does not alter the validity of the overall scientific conclusions of this study. In addition, the key outcomes are supported by other work since published by other laboratories. No other authors were involved in the provision of the fabricated data. As such the removal of this data does not undermine the findings of the paper or the integrity of the other authors.

Due to the removal of Figure 4**—**figure supplement 1 from this study, and according to the NHMRC and ICMJE guidelines defining the role of author and contributor, Professor Smyth’s name has been removed from the author list. Prof Smyth’s remaining contributions are now referred to in the acknowledgements section of the paper.

**Corrected author list:**

Jeroen Overman, Frank Fontaine, Mehdi Moustaqil, Deepak Mittal, Emma Sierecki, Natalia Sacilotto, Johannes Zuegg, Avril AB Robertson, Kelly Holmes, Angela A Salim, Sreeman Mamidyala, Mark S Butler, Ashley S Robinson, Emmanuelle Lesieur, Wayne Johnston, Kirill Alexandrov, Brian L Black, Benjamin M Hogan, Sarah De Val, Robert J Capon, Jason S Carroll, Timothy L Bailey, Peter Koopman, Ralf Jauch, Matthew A Cooper, Yann Gambin, Mathias Francois

**Originally published author list:**

Jeroen Overman, Frank Fontaine, Mehdi Moustaqil, Deepak Mittal, Emma Sierecki, Natalia Sacilotto, Johannes Zuegg, Avril AB Robertson, Kelly Holmes, Angela A Salim, Sreeman Mamidyala, Mark S Butler, Ashley S Robinson, Emmanuelle Lesieur, Wayne Johnston, Kirill Alexandrov, Brian L Black, Benjamin M Hogan, Sarah De Val, Robert J Capon, Jason S Carroll, Timothy L Bailey, Peter Koopman, Ralf Jauch, Mark J Smyth, Matthew A Cooper, Yann Gambin, Mathias Francois

**Corrected text:**

“The lack of inhibition of Sm4 on the primary tumor growth (Figure 4E) suggests that a potential combined effect between the drug treatment and surgery-induced inflammation is unlikely to be responsible for the increased survival, given that surgery is required on day 0 to inoculate the xenograft cancer cells into the mammary fat pad.”

Updated acknowledgement: “Professor Mark Smyth provided technical comments on the manuscript.”

**Original text:**

“To rule out any contribution by an inflammatory response as a result of surgery, we replicated the study by performing a sham surgery, without excising the tumor (Figure 4—figure supplement 1A, B). This approach confirmed that during the post-surgical period, primary tumor growth was unperturbed by Sm4 treatment and demonstrated that the combined effects of Sm4 with surgery-induced inflammation is unlikely to be responsible for the increased survival.”

The originally published Figure 4-figure supplement 1 is shown below for record:

**Figure 1:**
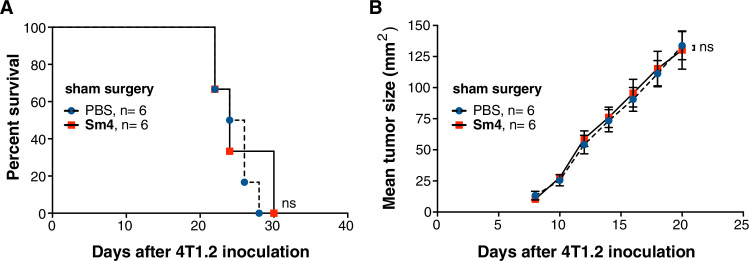


The article has been corrected accordingly.

